# miR-3156-3p is downregulated in HPV-positive cervical cancer and performs as a tumor-suppressive miRNA

**DOI:** 10.1186/s12985-017-0695-7

**Published:** 2017-02-04

**Authors:** Yu-Fang Xia, Gui-Hua Pei, Ning Wang, Yan-Ci Che, Feng-Sheng Yu, Fu-Fen Yin, Hai-Xia Liu, Bing Luo, Yan-Kui Wang

**Affiliations:** 1 Department of Obstetrics and Gynecology, The Affiliated Hospital of Qingdao University, Qingdao, China; 2 Department of Obstetrics and Gynecology, The Third Hospital of Qingdao, Qingdao, China; 3 Department of Medical Microbiology, Qingdao University Medical College, Qingdao, 266021 China

**Keywords:** MicroRNAs, Cervical cancer, Human papillomavirus, SLC6A6

## Abstract

**Background:**

Cervical cancer (CC) is the second most common cancer in females in developing countries. The two viral oncoproteins E6 and E7 mediate the oncogenic activities of high-risk human papillomavirus (HR-HPV), and HR-HPV, especially HPV16 or/and HPV18 (HPV16/18) play critical roles in CC through different pathways. microRNAs (miRNAs) may be associated with CC pathogenesis. Researches have indicated that human papillomavirus (HPV) may regulate cellular miRNA expression through viral E6 and E7. Herein, the purposes of this study were to identify the relationship between HPV infection and aberrantly expressed miRNAs and to investigate their pathogenic roles in CC.

**Methods:**

miRNA expression was assessed using a microRNAs microarray in HPV16 E6- and E7-integrated HPV-negative HT-3 cell lines and mock vector-transfected HT-3 cells. The microarray results were validated, and the expression of miR-3156-3p was identified in HPV-positive and -negative CC cell lines as well as primary CC and normal cervical epithelium tissues using quantitative reverse-transcription polymerase chain reaction (qRT-PCR). Cell Counting Kit-8 (CCK8), flow cytometry, transwell analysis, tube formation, and Western blotting were used to identify the functional role of miR-3156-3p in CaSki, SiHa, and HeLa cell lines.

**Results:**

Six underexpressed microRNAs (miR-3156-3p, 6779-3p, 4779-3p, 6841-3p, 454-5p and 656-5p) were consistently identified in HPV16 E6- and E7-integrated HT-3 cells. Further investigation confirmed a significant decrease of miR-3156-3p in HPV16/18 positive CC lesions. CCK8, flow cytometry, transwell analysis, tube formation assays, and Western blotting of the CC cell lines with miR-3156-3p over/under-expression in vitro showed that miR-3156-3p was involved in cell proliferation, apoptosis, migration, neovascularization, and SLC6A6 regulation.

**Conclusions:**

Our findings indicate that miR-3156-3p plays a suppressor-miRNA role in CC and that its expression is associated with HR-HPV infection.

## Background

CC is the second most common cancer and third leading cause of cancer death in females in less developed countries,with nearly 90% of CC deaths occurred in developing parts of the world [1]. HPVs are considered to be the major etiologic contributor to the development of CC and have been associated with 99.7% of cervical carcinomas [2]. HPVs are a family of small double-stranded circular DNA viruses with genomes containing 8-kb DNA sequences. So far, more than 150 types of HPVs have been identified. Among them, HR-HPV types 16 and 18 are the most prevalent and are responsible for 70% of CC [3]. E6 and E7 HR-HPVs are viral oncoproteins that inactivate p53 and pRB, respectively, and suppression of these two major cellular tumor suppressors subsequently contributes to cervical carcinogenesis.

miRNAs are small non-coding RNAs that are approximately 20 nucleotides in length and may regulate thousands of mRNA targets. miRNAs are transcribed in the nucleus and become associated with the RISC complex after processing. They primarily act as negative regulators of gene expression by binding to their complementary mRNA targets and either repressing translation or promoting mRNA degradation. Many studies indicate that changes in the expression of miRNAs may be associated with a variety of human cancers. These abnormally expressed miRNAs affect the expression of various oncogenic or tumor suppressor proteins that, in turn, alter cellular growth, invasion, and the metastatic potential of CC cells [4]. miRNAs can function in two opposing roles by either behaving as oncogenes or tumor suppressors depending on the tissue type and presence of specific targets [5]. To date, mounting evidence indicates that HPVs may regulate cellular miRNA expression through viral E6 and E7 [6].

To investigate the association between abnormal miRNA expression and HR-HPV infection in CC, we identified miRNA expression profiles by microarray in HPV16-E6 and -E7 integrated HPV-negative HT-3 cells and mock infection negative controls. The microarray results, validated by qRT-PCR, showed that miR-3156-3p expression was remarkably decreased in HPV16-E6 or/and -E7 integrated HPV-negative HT-3 and C-33A cell lines. Then, we examined the level of miR-3156-3p expression by qRT-PCR in 90 cases of human cervical tissues, including normal cervical epithelium and HPV-positive and HPV-negative cervical cancer tissues. We also detected a miR-3156-3p effect on the carcinogenetic processes in the CC cell lines. In addition, we validated a predicted target gene, SLC6A6, for miR-3156-3p with in vitro experiments and CC tissues. The aim of our study was to explore the role and mechanism of miR-3156-3p during cervical carcinogenesis induced by HR-HPV infection.

## Results

### Identification of miR-3156-3p as an aberrantly expressed miRNA in HR-HPV infected CC

**Fig. 1:**
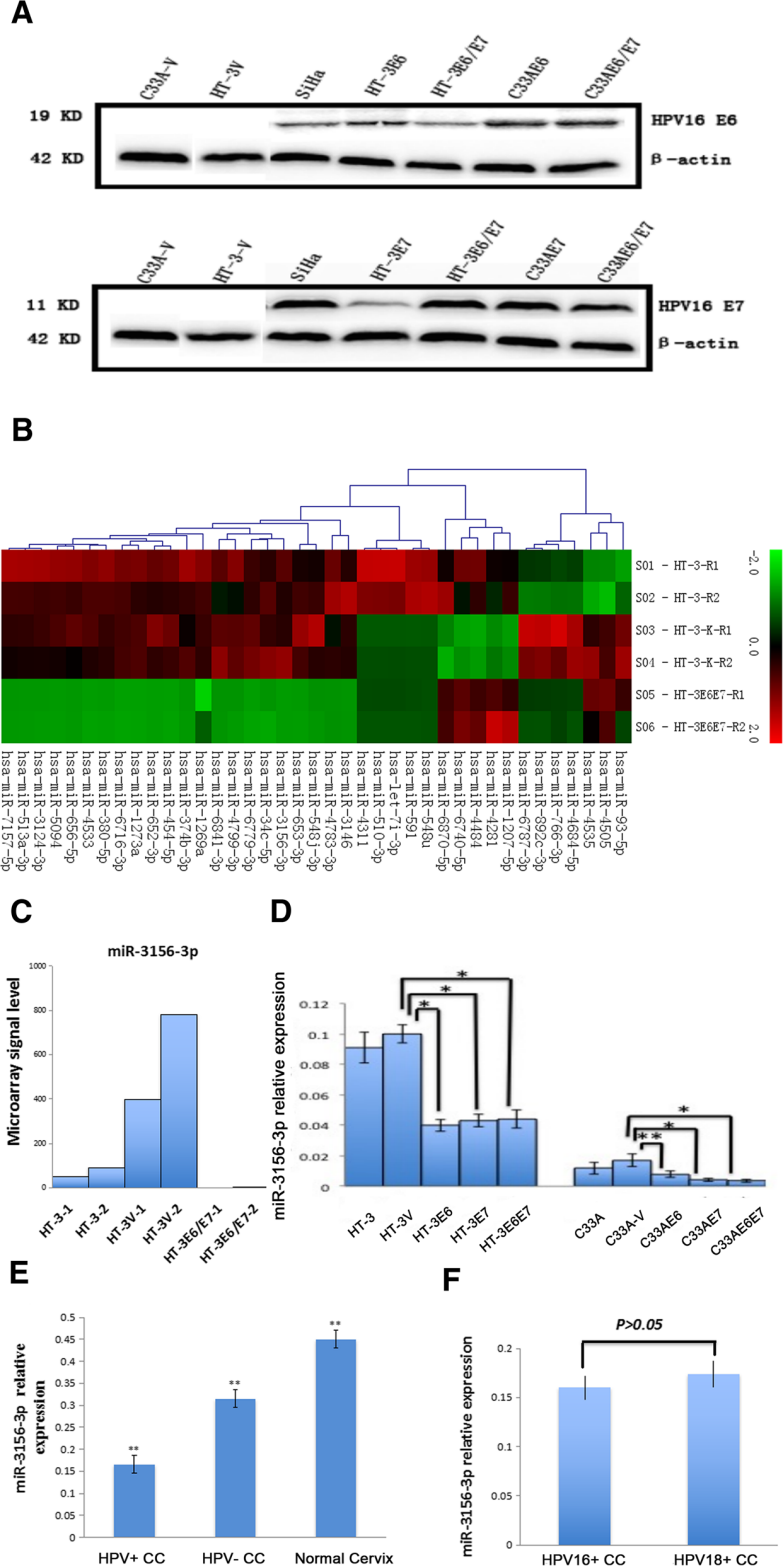
miR-3156-3p was aberrantly expressed in HR-HPV infected cervical cancer. **a** The transfection efficiency was tested by western blotting, which revealed that the transfected cells successfully expressed the E6, −E7, or -E6/E7 proteins. **b**-**c** From the microarray results, miR-3156-3p was selected as an aberrantly expressed miRNA in HPV16 E6- and E7-integrated HT-3. **d** The downregulation of miR-3156-3p was validated in HPV16 E6- and E7-integrated HT-3 and C-33A cells using qRT-PCR. **e** miR-3156-3p expression in clinical samples was evaluated using qRT-PCR. **f** miR-3156-3p expression in HPV16-positive and HPV18-positive clinical samples were evaluated using qRT-PCR. RNU6 served as the endogenous control for miRNAs. (**p < 0.01, *p < 0.05)

The transfection efficiency was tested by western blotting, which revealed that the transfected cells successfully expressed the E6, −E7, or -E6/E7 proteins (Fig. 1a). Using a microRNA microarray analysis, six downregulated miRNAs (miR-3156-3p, 6779-3p, 4779-3p, 6841-3p, 454-5p and 656-5p) were consistently found in HT-3E6/E7 cells compared to HT-3 V cells (Fig. 1b and c). A qRT-PCR analysis showed that miR-3156-3p was underexpressed in HPV16 E6- and E7-integrated HT-3 and C-33A cells. In HPV16 E6- and E7-integrated HT-3 cells, including HT-3E6, HT-3E7, and HT-3E6/E7, the level of miR-3156-3p was significantly lower than the level in HT-3 V. Yet, there was no difference between HT-3E6, HT-3E7, and HT-3E6/E7, as shown in Fig. 1d. A similar result was found in the C-33A cell line (Fig. 1d).

In addition, we examined the level of miR-3156-3p expression in normal cervical tissues and CC tissues, including 10 HPV-negative cases and 40 HPV16/18 positive cases. The results showed that miR-3156-3p expression was reduced in CC tissues compared to expression in normal cervical tissues (Fig. 1e). Furthermore, miR-3156-3p expression was lower in HPV16/18 positive cervical cancer than expression in HPV-negative lesions (Fig. 1e). However, there was no significant difference between HPV16-positive CC tissues (n = 26) and HPV18-positive CC tissues (n = 14) (Fig. 1f). Our findings suggest that miR-3156-3p probably contributes to cervical carcinogenesis and its reduction of miR-3156-3p expression in cervical cancer might be associated with HR-HPV infection.

### Altered miR-3156-3p expression influenced apoptosis, migration, invasion and tube formation of CC cells

**Fig. 2:**
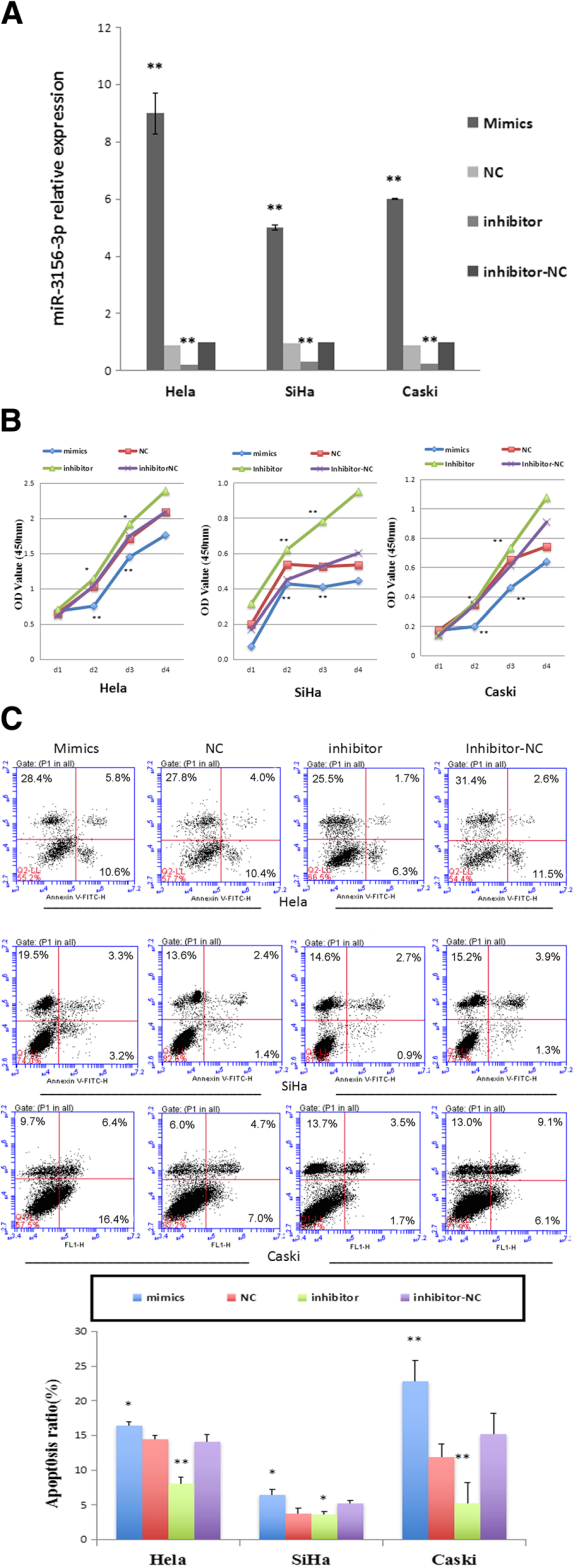
miR-3156-3p influenced cervical cancer cells proliferation and apoptosis. **a** The up-regulation and down-regulation of miR-3156-3p were confirmed using qRT-PCR in Hela, SiHa, Caski Cells transfected with mimics, inhibitor and corresponding negative controls (scrambled miRNAs). RNU6 served as the endogenous control for miRNAs. **b** Effect of miR-3156-3p mimics and inhibitor on HeLa, SiHa and Caski cell proliferation in a CCK8 assay. **c** Effect of miR-3156-3p mimics and an inhibitor on HeLa, SiHa and Caski cell apoptosis detected with flow cytometry. (**p < 0.01 vs. controls, *p < 0.05 vs. controls)

To delineate the role of miR-3156-3p in cervical carcinogenesis, we modulated miR-3156-3p expression by transient transfection with mimics and an inhibitor. The up-regulation and down-regulation of miR-3156-3p in Hela, SiHa, Caski Cells were confirmed using qRT-PCR (Fig. 2a). We examined the influence of miR-3156-3p on cell growth, apoptosis, migration, invasion and tube formation in CC cell lines.

First, we measured cellular proliferation using a CCK8 assay after cells were transfected with miR-3156-3p mimics or an inhibitor for 24, 48, 72 and 96 h. No significant difference in the proliferation rate was observed in Hela, Siha and Caski cells 24 h after transfection. However, 48 h after transfection with miR-3156-3p mimics, we noticed the rate of growth were 30%, 21% and 42%, respectively, less than that in NC group in Hela, Siha and Caski cells, (Fig. 2b). Conversely, after 24 h and 48 h transfections with and miR-3156-3p inhibitor, cellular proliferations were significantly higher than that in inhibitor-NC group in those 3 cell lines (Fig. 2b). More specifically, downregulation of miR-3156-3p resulted in a 13%, 38% and 5% increase in the cellular proliferation rate at 48 h and led to a 10%, 40% and 22% increase at 72 h in Hela, Siha and Caski cells, respectively (Fig. 2b).

Furthermore, the apoptosis rates of Hela, SiHa and Caski cells transfected with miR-3156-3p mimics or an inhibitor were examined using Annexin-V-FITC and a PI assay. As Fig. 2c shows, the apoptosis rate at 48 h after transfection significantly increased in Hela, Siha and Caski cells transfected with miR-3156-3p mimics and decreased in CC cells transfected with a miR-3156-3p inhibitor compared to the corresponding negative controls (all *p* < 0.05).

**Fig. 3:**
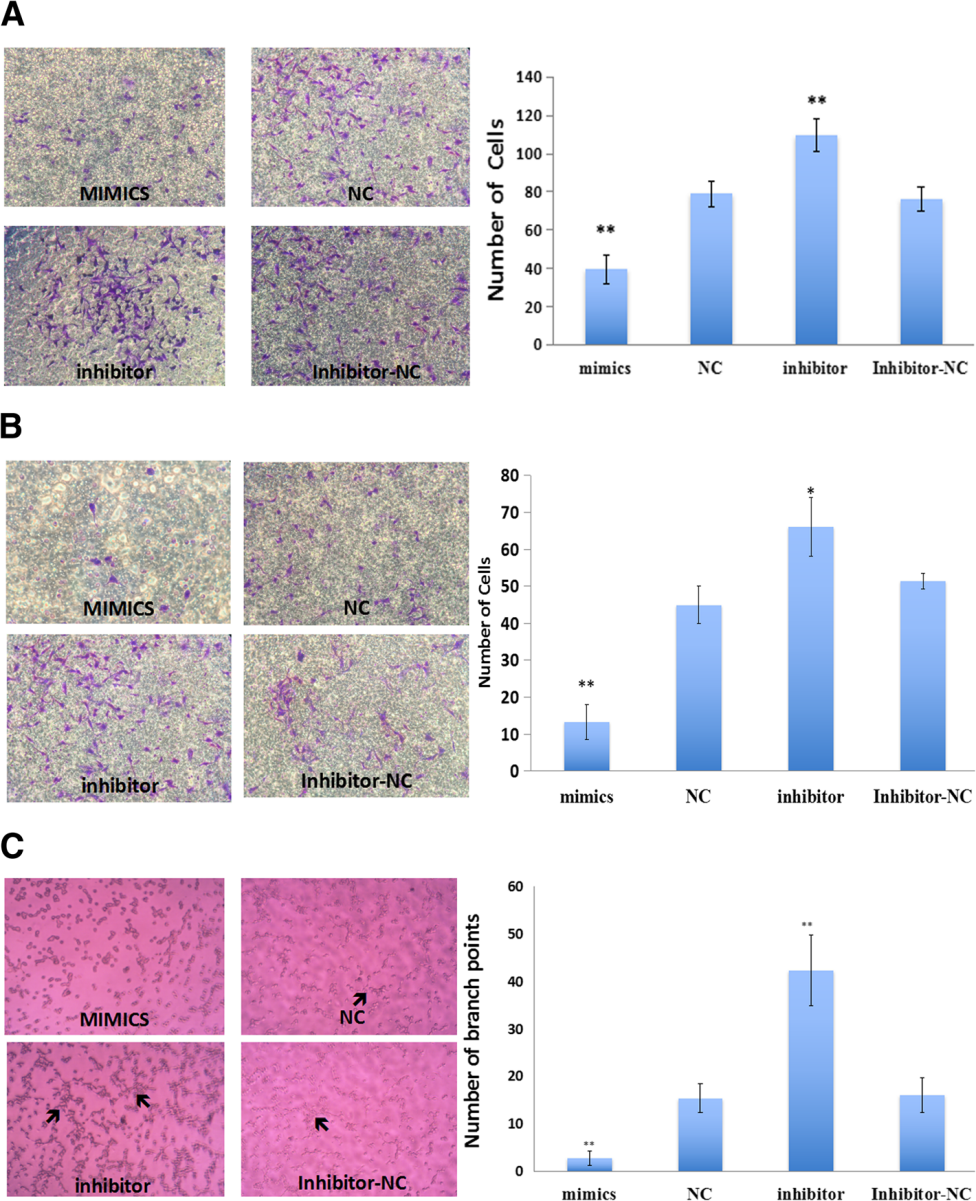
miR-3156-3p influenced cervical cancer cell migration, invasion and tube formation. The results showed that migration (**a**), invasion (**b**) and tube formation (**c**) significantly increased in the inhibitor group and decreased in the mimics group compared to the negative control groups. Arrow indicates tubular structure. (**p < 0.01 vs. controls, *p < 0.05 vs. controls)

To demonstrate that miR-3156-3p participates in the regulation of migration and invasion in CC, we used Caski cells that were transfected with miR-3156-3p mimics or an inhibitor to assess cell migration and invasion using the transwell chamber assay. The results showed that migration and invasion significantly increased in the inhibitor group and decreased in the mimics group compared to the negative control groups (*p* < 0.01 vs control, Fig. 3a and b).

To assess the capacity of miR-3156-3p on CC cells to mimic a vasculature network on Corning Matrigel, Caski cells were seeded on three-dimensional matrices after a 48 h transfection. We found that Caski cells in the inhibitor group were able to form several tubular structures, although the structures were less organized (Fig. 3c). In contrast, miR-3156-3p mimics completely abrogated the ability of Caski cells to form capillary structures (Fig. 3c).

Overall, our data indicates that miR-3156-3p modulates CC cell proliferation, apoptosis, migration, invasion and vasculogenic activity.

### miR-3156-3p negatively regulated SLC6A6 expression at the post-transcriptional level

**Fig. 4:**
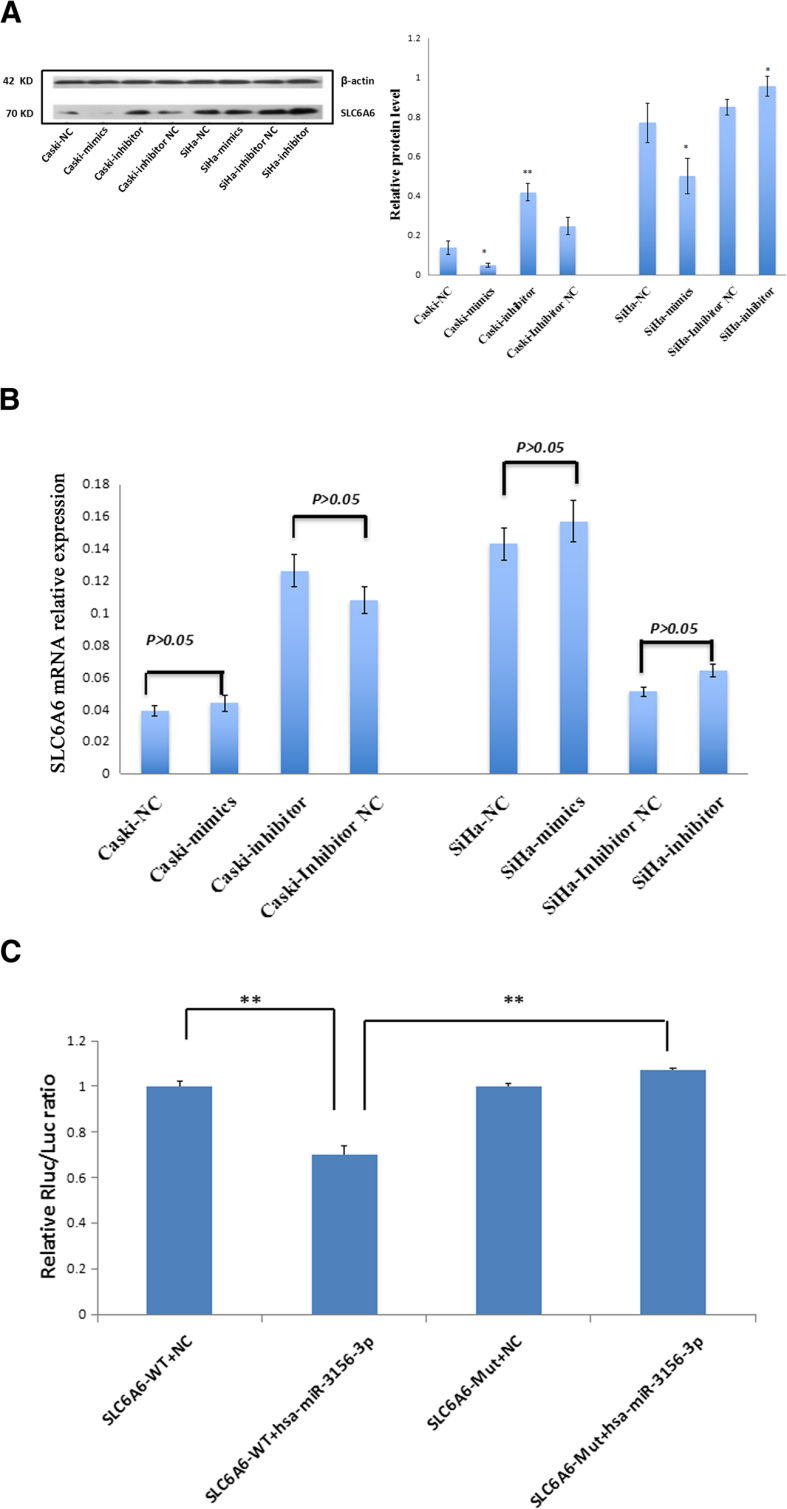
miR-3156-3p downregulates SLC6A6 expression at the post-transcriptional level in cervical cancer cells. **a** Protein level of SLC6A6 was detected by Western blot in Caski and SiHa cells transfected with miR-3156-3p inhibitor and mimics along with corresponding negative controls, respectively. **b** SLC6A6 mRNA level was examined by qRT-PCR in Caski and SiHa cells transfected with a miR-3156-3p inhibitor, mimics and corresponding controls. **c** HEK293 cells were co-transfected with miR-3156-3p and WT or Mut SLC6A6 3’UTR luciferase reporter construct. (**p < 0.01 vs. controls, *p < 0.05 vs. controls)

SLC6A6 was selected as a predictive target of miR-3156-3p through TargetScan, PicTar and miRBase software. The effect of miR-3156-3p on SLC6A6 mRNA and protein expression was tested in both Caski and SiHa cells using qRT-PCR, Western blot analysis, and transfection with a miR-3156-3p inhibitor or mimics. We found that miR-3156-3p mimics significantly inhibited SLC6A6 protein expression in both SiHa and Caski cells (Fig. 4a), whereas downregulation of miR-3156-3p caused a higher expression of SLC6A6 protein in Caski cells at 72 h post-transfection (Fig. 4a). There were no significant changes in the mRNA levels of SLC6A6 (Fig. 4b). Our findings suggest that SLC6A6 is negatively regulated by miR-3156-3p at the post-transcriptional level in CC cells.

To confirm that SLC6A6 was directly inhibited by miR-3156-3p, a dual-luciferase reporter system was used. We found that miR-3156-3p mimics markedly inhibited the firefly luciferase reporter activity of the wild-type SLC6A6 3’-UTR, but did not change the activity of the mutant 3’-UTR constructs (Fig. 4c). The results suggest that miR-3156-3p inhibited SLC6A6 expression by binding to the SLC6A6 3’- UTR.

### The expression of SLC6A6 increased in CC tissues

**Fig. 5:**
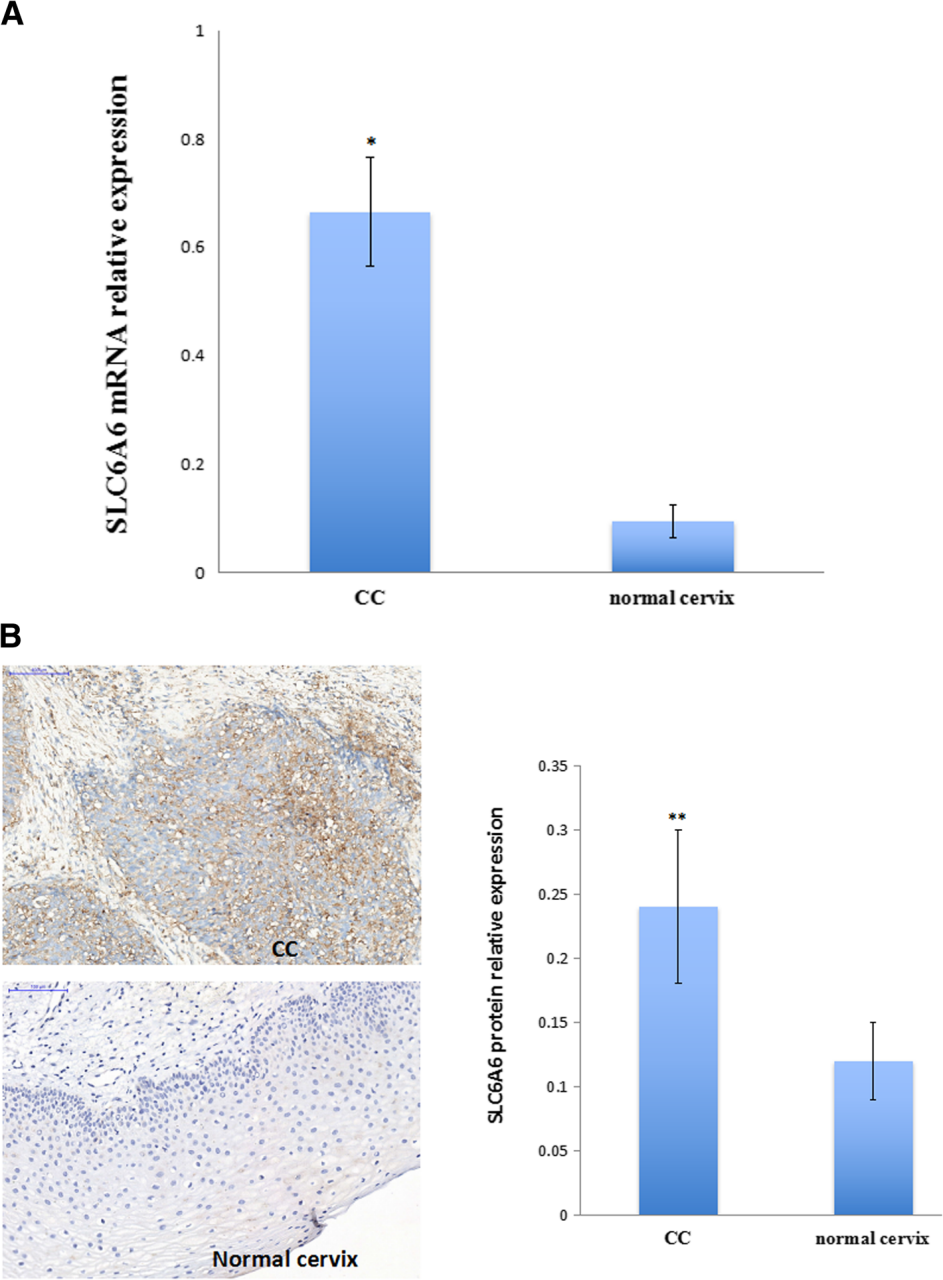
SLC6A6 expression in cervical cancer tissues. **a** SLC6A6 mRNA level was examined by qRT-PCR in cervical cancer and normal cervical tissue. **b** Immunohistochemical staining of the anti-SLC6A6 antibody in cervical cancer and normal cervical tissues. Strong SLC6A6 immunoreactivity was found in both the cytomembrane and cytoplasm in tumor tissues but not in the normal cervix tissues (scale bar, 100 μm). (**p < 0.01, *p < 0.05)

SLC6A6 mRNA and protein expression levels were tested in 40 HPV-positive CC tissues and 40 normal cervical tissues with qRT-PCR and immunohistochemical staining. As shown in Fig. 5a-b, the SLC6A6 mRNA level was significantly higher in HPV-positive CC samples compared to normal cervical samples (Fig. 5a). In immunohistochemical analysis, the positive expression of the SLC6A6 protein was primarily localized to the cell membrane. As shown in Fig. 5b, SLC6A6 protein expression was markedly higher in CC compared to normal cervix tissue.

**Fig. 6:**
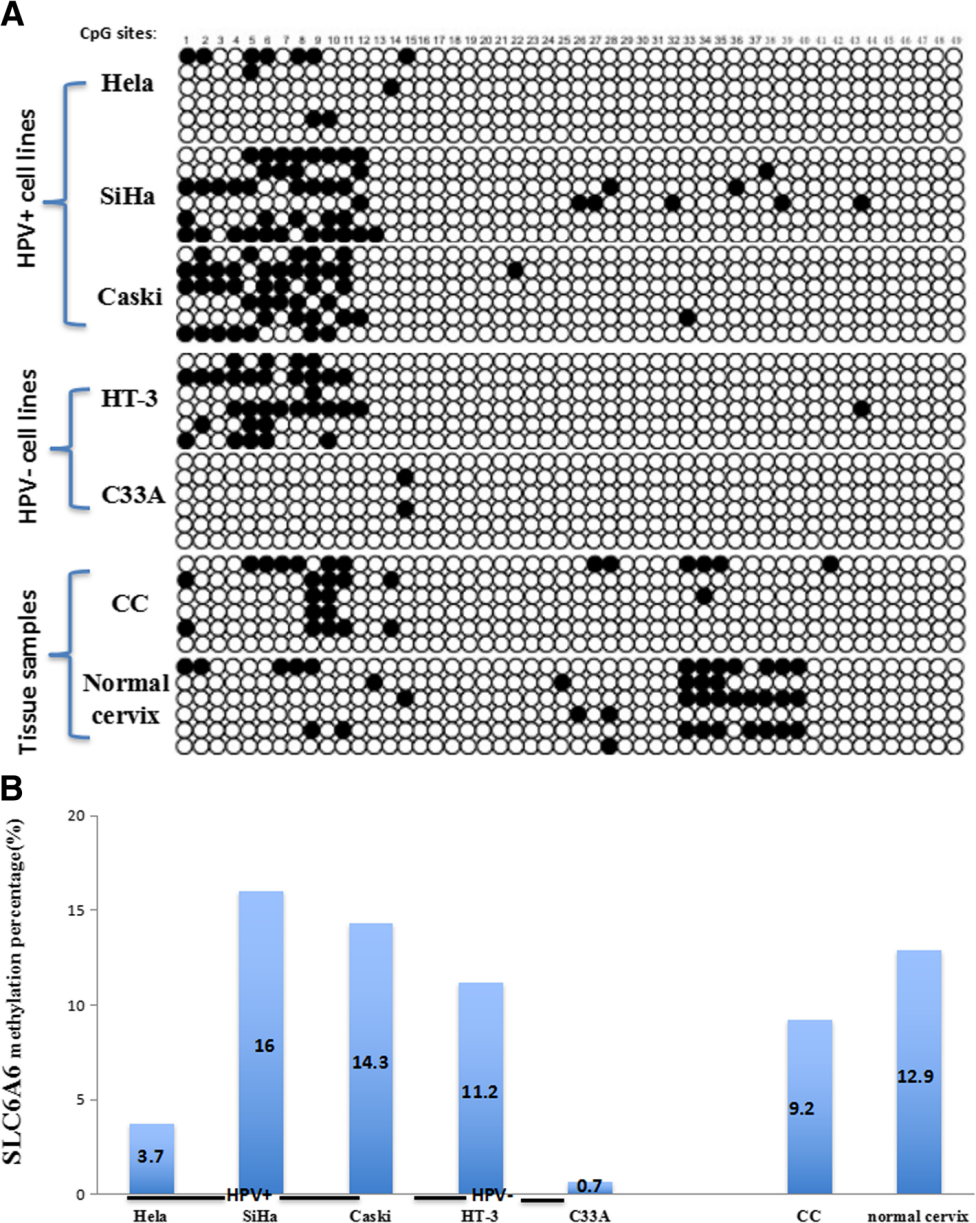
Promoter methylation of SLC6A6 in cervical cancer cell lines and tissue samples detected with BGS. **a** BGS analysis of the SLC6A6 promoter in CC cell lines. Each circle is one CpG site and filled circles are methylated CpG sites. There are 49 CpG sites in the BGS region. **b** The SLC6A6 methylation rate in cervical cancer cell lines and tissue samples. The methylation ratio of Hela, SiHa, Caski, and HT-3 cells as well as cervical cancer tissues and normal cervix tissues was 3.7%, 16.0%, 14.3%, 11.2%, 0.7%, 9.2% and 12.9%, respectively

DNA methylation is another form of epigenetic modification. To evaluate whether DNA methylation was involved in SLC6A6 gene expression in CC, a BGS assay was performed to examine the methylation status of the promoter region of SLC6A6 in HPV-positive and HPV–negative CC cell lines, including Hela, SiHa, Caski, HT-3 and C-33A cells, as well as CC samples and normal cervical samples. As shown in Fig. 6a, 49 individual CpG sites within CpG island regions were sequenced to identify methylated cytosine residues. The results of SLC6A6 promoter methylation showed no significant differences between CC samples and normal cervical samples(9.2% vs. 12.9%). SLC6A6 promoter hypomethylation was also found in both HPV-positive Hela, SiHa and Caski cells and HPV-negative HT-3 and C33A cells (Fig. 6b).

## Discussion

Persistent human papillomavirus infection is the main etiological agent in CC initiation and progression [7]. The central core of HPV E6 and HPV E7 is the binding and inactivation of the tumor suppressor proteins p53 and pRB, respectively, which affect the molecular pathways involved in cervical carcinogenesis [8]. However, HPV is a necessary- but not sufficient- cause for cervical carcinogenesis [7]. Recently, accumulating evidence has shown that aberrant expression of cellular oncogenic and tumor suppressive miRNAs have an important role in cervical carcinogenesis. Using a miRNA microarray, we found that miR-3156-3p expression was downregulated significantly in HPV16 E6- and E7-integrated HT-3 cells compared to a negative control. Using a qRT-PCR assay, we confirmed miR-3156-3p expression was downregulated in HPV16 E6- and E7-stable-transfected HT-3 and C33A cell lines. miR-3156-3p is the mature sequence of miR-3156-1 and miR-3156-2, which are located on chromosomes 10 and 28, respectively. miR-3156-3p annotations in miRbase came from a deep-sequencing analysis of breast cancer in 2010 [9]. However, the function of miR-3156-3p is still unknown. In this study, we identified miR-3156-3p expression in normal cervical epithelium, HPV-negative and HPV16/18-positive CC using RT-PCR. Then, reduced miR-3156-3p expression was found in CC tissues and its expression in HPV-positive tumors was the lowest among three groups. We therefore presumed that reduction of miR-3156-3p expression was involved in cervical carcinogenesis induced by HR-HPV infection.

To study the functional role of miR-3156-3p in CC, we modulated miR-3156-3p expression in CC in vitro using miRNA mimics and an inhibitor. We found that upregulation of miR-3156-3p expression distinctly inhibited cell growth and increased cell apoptosis in HPV18-positive Hela cells and HPV16-positive SiHa and Caski cells. Conversely, downregulation of miR-3156-3p expression remarkably promoted cell growth and decreased cell apoptosis in all three cell lines. Furthermore, our results clearly demonstrated that miR-3156-3p significantly suppressed immigration and invasion in Caski cells. Some recent studies implicate miRNAs in the regulation of various aspects of angiogenesis [10, 11]. Vascular mimicry is thought to foster cancer progression by contributing to the delivery of a nutrient supply to starved tumors and increasing cancer cell dissemination [12]. In this study, we found that transfection with miR-3156-3p mimics resulted in significant impairment of tube-forming activity in Caski cells. Thus, our combined results suggest that miR-3156-3p acts as an inhibitor of cervical cancer tumorigenesis.

Mechanisms by which microRNAs can regulate gene expression are still not fully understood, including messenger RNA degradation, translation inhibition, promoter binding, protein binding, or direct interaction with other non-coding RNAs [13]. It is now well known that abnormally expressed miRNA primarily functions as a negative regulator of target gene expression through full or partial complementary binding to the 3’-UTR, which leads to mRNA cleavage or mRNA translation repression [14]. In the present study, a bioinformatics search for potential target genes of miR-3156-3p was performed using 3 common databases, and SLC6A6 was identified as a possible target. Dual luciferase reporter gene activity confirmed that miR-3156-3p could target the 3’-UTR of SLC6A6 directly. From Western blot analysis of the CC cells with miR-3156-3p overexpression or underexpression, SLC6A6 consistently had a negative correlation with the expression of miR-3156-3p at the protein level but not the mRNA level.

SLC6A6 (also referred to as TauT) is a high-affinity, low-capacity, multi-pass membrane protein that transports taurine and β-alanine in a Na^+^- and Cl^−^-dependent manner [15]. SLC6A6 signaling has also been shown to affect cell proliferation and cell survival [16]. Han and colleagues demonstrated that SLC6A6 overexpression protects kidney cells against cisplatin-induced cell death through p53 activation [17, 18]. A recent study found that SLC6A6 plays an important role in the maintenance of side population cells and their cancer stem cell properties, including enhanced prosurvival activity, tumor initiation and chemoresistance in colorectal cancer [19]. Tastesen et al. showed that knockdown of TauT leads to a significant increase in apoptosis following cisplatin exposure and that cisplatin resistance correlated with increased TauT expression/activity [20]. Accordingly, Sørensen BH et al. found that acquired resistance in human ovarian A2780 cancer cells correlates with increased TauT activity [21]. Consistent with these reports, our study showed that both mRNA and protein expression levels of SLC6A6 were significantly higher in CC tissues compared to normal cervical tissues, and SLC6A6 may play an important role in cervical carcinogenesis. However, the regulation mechanism of SLC6A6 expression is still less understood and require further study. Related researches found SLC6A6 gene could be repressed by the p53 tumour suppressor gene and be transactivated by proto-oncogenes such as WT1, c-Jun, and c-Myb [17, 22]. Our findings suggest the expression of SLC6A6 was regulated by miR-3156-3p at post-transcriptional level in vitro. Additionally, SLC6A6 expression seems to be unaffected by methylation regulation. Meanwhile, the tumor-suppressor effect of miR-3156-3p may be due to the regulation of SLC6A6 in cervical cancer. Therefore, miR-3156-3p may be a promising therapeutic strategy for CC.

## Conclusions

In conclusion, our findings indicated that miR-3156-3p plays a tumor-suppressor role in CC and its downregulation is associated with CC pathogenesis through promotion of proliferation,migration,invasion and tube formation. miR-3156-3p may be a novel therapeutic strategy for the treatment of CC.

## Methods

### Clinical samples and HPV-DNA detection and genotyping

Forty HPV16/18-positive cervical cancer, 10 HPV-negative cervical cancer and 40 HPV16/18-negative normal cervical tissues were provided by the Affiliated Hospital of Qingdao University (Qingdao, China). The use of tissue specimens was approved by the Hospital Research Ethics Committee and informed consent was obtained from patients. All tissues were histologically and independently diagnosed by two pathologists, and CC was classified histopathologically according to the International Federation of Gynecology and Obstetrics (FIGO) staging system [23]. All tissue samples were immediately frozen in liquid nitrogen after surgical removal and stored at −70 °C until use.

Cervical HPV infection was detected with the presence of HPV DNA by PCR amplification using general primers sets MY09/MY11, GP5+/6+ and SPF1/2(RIBOBIO,Guangzhou, China) [24–26]. The HPV-detection results of the three methods were all consistent, and the negative samples were designated as HPV-negative samples. The positive samples were further tested with HPV-type-specific PCR for genotypes 16 and 18 [27, 28]. The PCR protocol details are available from the corresponding author.

### Cell lines and transfection

The HPV16/18-positive CC cell lines HeLa, CaSki, SiHa and the HPV-negative CC cells C33A and HT-3 were obtained from Shanghai Institute for Biological Sciences and the Chinese Academy of Sciences Institute of Cell Resource Center (Shanghai, China). In the present study, we established ectopically expressed HPV16 E6, −E7, and -E6/E7 cell models of C33AE6, C33AE7, C33AE6/E7, HT-3E6, HT-3E7, and HT-3E6/E7 by transfecting HPV16 E6, −E7, and -E6/E7 oncogenes with lentivirus vectors into the HPV-negative C33A and HT-3 cells in our lab. The C33A-vector (C33A-V) and HT-3 vector (HT-3 V) cells were established by transfecting C33A and HT-3 cells with lentivirus vectors that did not code for the HPV16 E6, −E7, or -E6/E7 proteins as controls. Stable transfections were treated with 10 μg/ml puromycin for 3 weeks. The transfection efficiency was tested with Western blotting, which revealed that the transfected cells successfully expressed E6, −E7, or -E6/E7 proteins. All cells were maintained in a humidified incubator set at 37 °C and 5% CO2. The miRNA expression profiles were identified in HT-3E6/E7, HT-3 V and HT-3 cell lines using an LC Sciences microRNA microarray(Hangzhou, China) containing 2578 human mature microRNAs based on Sanger miRBase Release 20.0. Differentially expressed miRNAs were identified by fold changes as well as by *p*-values that were calculated by *t*-test. The threshold that was set for up-regulated and down-regulated miRNAs was a fold change ≥4 and a *p* value <0.01.

### miR-3156-3p mimics and inhibitor

**Fig. 7:**
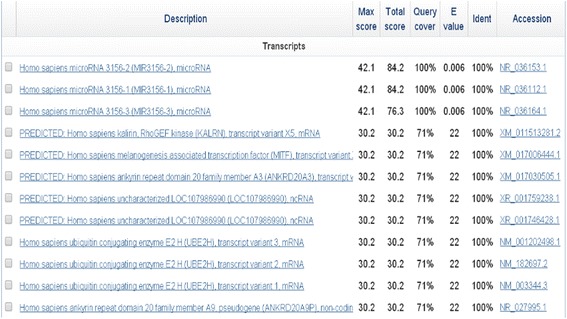
Sequence alignments of miR-3156-3p mimics and inhibitor were assessed using NCBI blast

miR-3156-3p mimics (chemically double-stranded oligonucleotides, 5′-CUC CCA CUU CCA GAU CUU UCU-3′), miR-3156-3p hairpin inhibitor (single-stranded chemically modified oligonucleotides, 5'-AGA AAG AUC UGG AAG UGG GAG-3') and corresponding negative controls were purchased from GenePharma (Shanghai, China). The result of blast analysis indicated the mimics and inhibitor were specific and potent to miR-3156-3p using NCBI blast (Fig. 7). Negative controls were a random sequence which had been extensively tested in human cell lines and tissues and validated to not produce identifiable effects on known miRNA function. FAM dye-labeled negative controls had the same oligonucleotide sequence as unlabeled negative control and were used to monitor transfection efficiency. Transient transfections were performed when the cells reached 30-50% confluence using the RNAi-Mate transfection reagent (GenePharma, Shanghai, China) according to the manufacturer’s instructions. At the indicated times after transfection, the cells were harvested and used in experiments.

### RNA isolation and qRT-PCR for miR-3156-3p

SYBR Green-based real-time quantification of miRNAs was used to determine miR-3156-3p expression as previously described. Total RNA was extracted using the Trizol reagent (Invitrogen). The quality of total RNA is assessed by ultraviolet spectrophotometer, the total RNA ration of A260/A280 between 1.8 and 2.0 was considered as high quality. Then, 1 μg of total RNA was subsequently reverse-transcribed to cDNA with a miR-3156-3p-specific stem-loop-like RT primer(RIBOBIO,Guangzhou, China) following the manufacturer’s protocol. Then, qRT-PCR was performed using SYBR Green mix with primers specific to miR-3156-3p(RIBOBIO,Guangzhou, China). Small nuclear RNA RNU6 was used as an endogenous control. Relative quantification of the miRNA expression was calculated with the 2^-ΔΔCT^ method.

### qRT-PCR for mRNA

cDNAs were synthesized using a transcriptor first strand cDNA synthesis kit (Roche). Then, qRT-PCR for mRNA was performed using FastStart Universal SYBR Green Master (Roche). The primers used for qRT-PCR include, for SLC6A6, forward 5’- GCT TCC CGT ACC TCT GCT AC-3’ and antisense 5’-TGG CCT ATG ATG ATC TCC AA-3’. Glyceraldehyde 3-phosphate dehydrogenase (GAPDH) was used as an endogenous control. Relative quantification of the mRNA expression was calculated with the 2^-ΔΔCT^ method.

### Cell proliferation assay and apoptosis analysis

Cell proliferation was assessed with a Cell Counting Kit-8 (CCK-8) assay kit (Dojindo, Japan). Hela, Siha and Caski cells were separately cultured in 96-well plates overnight at a density of 5000 cells/well then transfected with miR-3156-3p mimics or an inhibitor as described above. At 1, 2, 3, 4 and 5 days after transfection, 10 μl of CCK8 solution was added to each well for 1 h and absorbance readings at 450 nm were obtained in triplicate using a spectrophotometric plate reader. The data were obtained from the measurement of 4 replicate wells for each data point.

For the apoptosis analysis, cells were harvested after transfection for 48 h by trypsinization, washed twice using cold PBS and were subsequently stained with Annexin V-FITC and propidium iodide using Annexin V apoptosis detection kit FITC (ebioscience,San Diego, CA 92121 USA) at room temperature, and apoptosis analysis was performed with FACS flow cytometry (BD Biosciences, Franklin Lakes, NJ).

### Transwell migration and invasion assay

Caski cells were harvested by trypsinization/EDTA. Aliquots of cells (1.5 × 10^5^) were placed into transwell chambers(Corning Incorporated, USA) for migration assay, and 1 × 10^4^ cells were placed into upper chambers coated with 150 mg Matrigel for invasion assay. The lower chambers were filled with DMEM containing 10% FBS. After incubation at 37 °C for 12–24 h, cells remaining on the upper surface of the membrane were removed. Cells on the lower surface of the membrane were fixed and stained with crystal violet. Then stained cells were visualized and counted under a light microscope. The assays were performed in triplicate.

### Tube formation assay

To assess tube formation, 50 μl Matrigel (Corning Incorporated,USA) was plated to 96-well plates at a horizontal level and incubated for 30 min at 37 °C. Then Caski cells after transfection for 24 h were harvested, re-suspended with serum-free DMEM and loaded on the top of the Matrigel at a density of 1.5 x 10^4^ cells per well. Each conditional group contained 4 wells. Following incubation at 37 °C for 12 h, each well was analyzed directly under a microscope. Under a microscope with 10x phase contrast, tubules in each field were imaged and an average of tubules from 3 random fields in each well was counted. The assays were repeated three times.

### Western blot analysis

Cells were washed twice with ice-cold PBS and treated with RIPA lysis buffer. Protein concentrations were quantified by the BCA protein assay kit (Beyotime, Haimen, China). Protein were separated on 8% SDS-PAGE gels, transferred onto PVDF membranes (Bio-Rad, Hercules, CA, USA) and blocked for 1 h at room temperature. Membranes were probed with primary antibodies anti-SLC6A6 (1:1000 dilution; Abcam, USA) at 4 °C overnight followed by incubation with HRP-conjugated secondary antibodies. Each sample was also treated with anti-β-actin antibody (Sigma-Aldrich) as a control. Blots were detected using an ECL detection system.

### Tissue samples immunohistochemistry

Paraffin-embedded tissue blocks were sectioned (4 μm) for immunohistochemical staining. After antigen retrieval and peroxidase blocking, the sections were incubated with rabbit polyclonal antibody against human SLC6A6 (1:50 dilution; Abcam, USA) at 4 °C overnight. After 3 washings in sterile phosphate-buffered saline, sections were incubated with a horseradish peroxidase (HRP)-conjugated antibody against rabbite immunoglobulin G (IgG) (Invitrogen, Carlsbad, Calif) (1 : 1000 dilution). Isotype-matched IgG control was used in each experiment. The percentage of positive cells was graded according to the following criteria: 0, less than 10%; 1, 10–30%; 2, 30–50%; or 3, more than 50%[29].

### Bisulfite genomic sequencing (BGS)

Genomic DNA was extracted from cells and tissue specimens using the phenol-chloroform method. Bisulfite treatment was performed using a CpGenomeTM Universal DNA Modification Kit (Millipore, USA), following the manufacturer’s instructions. Modified DNA was amplified, and PCR products were gel-purified and sub-cloned into a pTG19-T vector system (MAP BIOTHCH, China). Ten colonies were sequenced to assess the degree of methylation at each CpG site. The primers used for SLC6A6 PCR were listed: forward 5’-GGT AAG GTT AGG ATT TTG GAG TTT T-3’, antisense 5’-TCA ACA TCA CCC ATC CTA AAT A -3’.

### Dual-luciferase reporter assay system

For luciferase reporter, the 3’UTR of SLC6A6 containing the putative binding sites for miR-3156-3p (682 bp) or a mutant lacking the miR-3156-3p seed sequences was amplified by PCR and cloned into the pmiR-RB-REPORT vector to generate reporter constructs (RIBOBIO,Guangzhou, China). Constructs were verified by sequencing. Transfection of miR-3156-3p mimics was performed when HEK293 cells were grown to 50-70% confluence (Invitrogen, Carlsbad, CA, USA) according to the manufacturer’s instructions. Cells were harvested 48 h after co-transfection and assayed with the Dual Luciferase Assay kit (Promega, Madison, WI, USA) according to the manufacturer’s protocol.

### Statistical analysis

SPSS17.0 statistical software package was used for statistical analysis. Experiments were repeated independently at least three times, and the results are expressed as mean ± SD. Statistical differences between groups were evaluated using Student's paired two tailed *t*- test. *p* < 0.05 was considered statistically significant.
